# Binaural Diplacusis and Its Relationship with Hearing-Threshold Asymmetry

**DOI:** 10.1371/journal.pone.0159975

**Published:** 2016-08-18

**Authors:** David Colin, Christophe Micheyl, Anneline Girod, Eric Truy, Stéphane Gallégo

**Affiliations:** 1 Lyon Neuroscience Research Center, IMPACT Team, CRNL, INSERM U1028, CNRS UMR5292, Lyon, France; 2 Institut des Sciences et Techniques de la Réadaptation, Lyon, France; 3 Starkey France, Créteil, France; 4 Departement ORL, Hôpital Edouard Herriot, Centre Hospitalier et Universitaire, Lyon, France; 5 University Lyon 1, Lyon, France; Johns Hopkins University, UNITED STATES

## Abstract

Binaural pitch diplacusis refers to a perceptual anomaly whereby the same sound is perceived as having a different pitch depending on whether it is presented in the left or the right ear. Results in the literature suggest that this phenomenon is more prevalent, and larger, in individuals with asymmetric hearing loss than in individuals with symmetric hearing. However, because studies devoted to this effect have thus far involved small samples, the prevalence of the effect, and its relationship with interaural asymmetries in hearing thresholds, remain unclear. In this study, psychometric functions for interaural pitch comparisons were measured in 55 subjects, including 12 normal-hearing and 43 hearing-impaired participants. Statistically significant pitch differences between the left and right ears were observed in normal-hearing participants, but the effect was usually small (less than 1.5/16 octave, or about 7%). For the hearing-impaired participants, statistically significant interaural pitch differences were found in about three-quarters of the cases. Moreover, for about half of these participants, the difference exceeded 1.5/16 octaves and, in some participants, was as large as or larger than 1/4 octave. This was the case even for the lowest frequency tested, 500 Hz. The pitch differences were weakly, but significantly, correlated with the difference in hearing thresholds between the two ears, such that larger threshold asymmetries were statistically associated with larger pitch differences. For the vast majority of the hearing-impaired participants, the direction of the pitch differences was such that pitch was perceived as higher on the side with the higher (i.e., ‘worse’) hearing thresholds than on the opposite side. These findings are difficult to reconcile with purely temporal models of pitch perception, but may be accounted for by place-based or spectrotemporal models.

## Introduction

Binaural pitch diplacusis—also referred to as ‘binaural harmonic diplacusis’ or, in short, ‘binaural diplacusis’–refers to a perceptual anomaly, whereby the same tone is perceived as having a different pitch depending on whether it is presented in the left ear or the right ear of the same listener [1–11]. Results in the literature suggest that binaural diplacusis is more prevalent, and more pronounced, in hearing-impaired individuals, especially, individuals with asymmetric hearing loss, than in normal-hearing individuals [1–11]. This effect is of particular interest in the context of the long-standing debate in hearing science, between ‘place’ and ‘temporal’ theories of pitch, a fundamental perceptual attribute of sound [12]. According to place theories, pitch is encoded in the spatial (or ‘tonotopic’) pattern of excitation produced by sounds in the auditory system [13–15]. According to temporal theories, pitch is encoded in the temporal pattern of sound-evoked responses, specifically, in inter-spike intervals [15–17]. In the current state of knowledge, binaural diplacusis is, arguably, more readily explained based on a pure place code or a spectrotemporal code than based on a purely temporal code. In particular, neurophysiological studies have demonstrated shifts in the frequency corresponding to the peak of basilar-membrane displacement, or to the peak firing rate of auditory-nerve fibers, as a result of cochlear damage [18], which could be the origin of pitch shifts and binaural diplacusis in hearing-impaired individuals. By contrast, studies examining the impact of cochlear damage on inter-spike intervals at the level of single auditory-nerve fibers have, for the most part, found no effect [19–24], thus questioning a purely temporal explanation for binaural diplacusis.

In addition to being of theoretical interest, the existence of pitch discrepancies between the two ears may be relevant for understanding the perceptual difficulties experienced by hearing-impaired listeners. Such discrepancies may be indicative of a mismatch between the information received by the left and right ears, which may impact the combination of information across the two ears. In this context, it would be useful to know how prevalent this effect is in hearing-impaired individuals, and how its magnitude can be predicted based on audiometric measures such as hearing thresholds. Although, as mentioned above, binaural diplacusis appears to be more prevalent, and more pronounced, in individuals with unilateral or asymmetric hearing loss than in individuals with normal hearing [1–11], all previous studies of binaural diplacusis involved relatively small sample sizes (ten or fewer participants); because of this, estimates of its prevalence in hearing-impaired individuals, and of its statistical relationship with left/right asymmetries in hearing thresholds, remain largely uncertain. In particular, an open question is whether the sign and magnitude of the pitch discrepancy between the left and right ears can be predicted using the sign and magnitude of the difference in hearing thresholds between the left and right ears.

In this study, binaural diplacusis was assessed in 55 participants, including 43 individuals with high-frequency hearing-loss of presumed cochlear origin, and 12 individuals with normal hearing thresholds. In the hearing-impaired (HI) participants, binaural diplacusis was assessed at two frequencies, 500 Hz and a higher frequency close to the audiogram ‘cutoff’–defined as the point where the slope of the audiogram became steeper. Based on previous findings, the following predictions were made. Firstly, binaural diplacusis (defined as a statistically significant difference in pitch for a tone of the same frequency across the left and right ears) should be more prevalent, and the size of the pitch difference between the left and right ears should be larger, in individuals with hearing loss than in individuals with normal hearing. Secondly, the magnitude of the pitch difference between the left and right ears should tend to increase with the magnitude of the asymmetry in hearing thresholds. Thirdly, in individuals with asymmetric hearing thresholds, for a given stimulus frequency, the pitch should be higher in the ear with the larger (i.e., ‘worse’) hearing thresholds. Fourthly, all other things being equal, binaural diplacusis should be less prevalent, and less marked, at 500 Hz than at higher frequencies. The aim of this study was to test these predictions. Results were consistent with the first three predictions, but not with the latter.

## Methods

### Subjects

Twelve (6 female; 6 male) normal-hearing (NH) and 43 (18 female; 25 male) HI individuals were tested. The NH participants were aged between 21 and 52 years (mean = 32.1 years; SD = 12.5 years). The HI participants were aged between 42 and 79 years (mean = 65.4 years; SD = 8.34 years). All HI participants had hearing loss sloping toward high frequencies. For about half (20) of these participants, the hearing loss was asymmetric across the two ears, with an interaural difference in absolute thresholds of at least 15 dB at the audiogram ‘cutoff frequency’ (Fc) in the participant’s ‘better’ ear, i.e., the ear with lower average absolute thresholds, where the average was computed across the following audiometric frequencies: 500, 1000, 2000, and 4000 Hz. The audiometric frequencies tested were: 250, 500, 1000, 1500, 2000, 3000, 4000, 6000, and 8000 Hz. To determine Fc, we computed the audiogram slope per octave. For most patients, Fc was determined as the lowest of the two consecutive audiometric test frequencies for which the slope of the audiogram (absolute thresholds as a function of frequency) was higher than 10 dB. For 8 patients who had an almost flat audiogram, and in whom the audiogram slope was never higher than 10 dB, Fc was set to 2000 Hz, the mode of the statistical distribution of Fc across HI participants for whom Fc could be determined using the audiogram slope-difference criterion.

**Fig 1:**
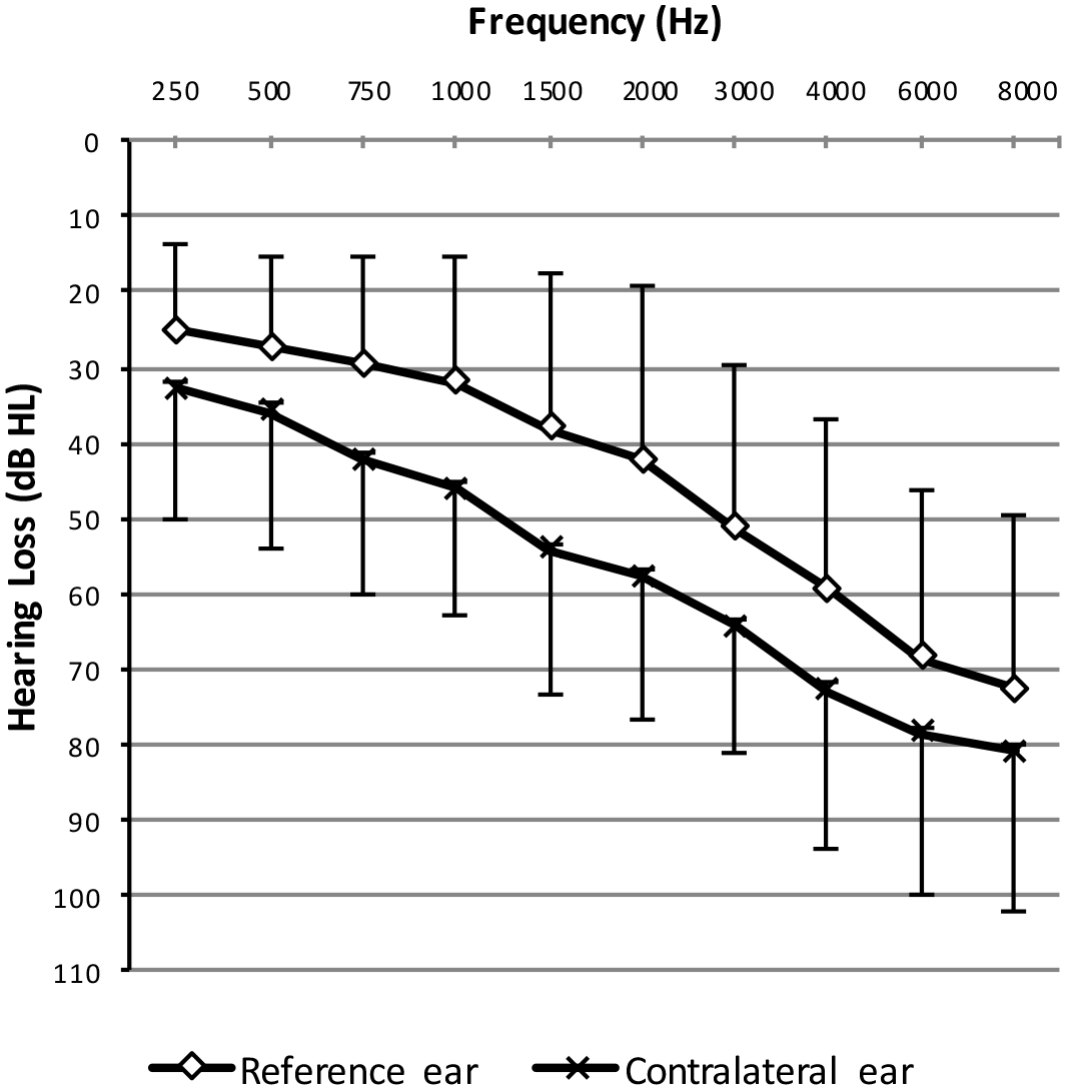
Mean hearing loss as a function of frequency for the HI participants. Green: mean hearing loss for the ‘better’ ear; purple: mean pure-tone thresholds for the contralateral ear. Error bars show +/- 1 standard deviation (SD).

Fig 1 shows the mean hearing loss a function of frequency across all HI participants for the ‘better’ ear and the contralateral (‘worse’) ear. The average hearing loss across 500, 1000, 2000, and 4000 Hz was 40 dB HL ± 15.8 dB (standard deviation, SD) for the better ear, and 53 dB HL ± 15.5 dB (SD) for the contralateral ear.

The study was approved by the institutional review board of the Institut de Sciences et Techniques de Readaptation, Université Lyon I, and was performed in accord with the Declaration of Helsinki. All participants provided written informed consent.

### Procedure

Each participant was tested individually as follows. Firstly, pure-tone hearing thresholds were measured using the procedure described in [25]. Hearing thresholds were measured at 250, 500, 1000, 1500, 2000, 3000, 4000, 6000, and 8000 Hz. Secondly, maximum comfort levels (MCL) were measured using the following procedure. The participant was informed that this test sought to measure the maximum sound level that he/she could tolerate, and that he/she should indicate when the sound presented was becoming uncomfortably loud. Initially, a 1000-Hz pure-tone was presented with a level equal to the higher of 60 dB HL, or the hearing threshold plus 5 dB. If the participant indicated that the loudness was acceptable, the level was increased by 5 dB. This was repeated until the participant indicated that sound was becoming uncomfortably loud, at which point the next frequency was tested. Besides 1000 Hz, the following frequencies were tested in random order: 250, 500, 1000, 2000, 3000, and 4000 Hz. The hearing thresholds and MCLs were measured separately in the left and right ears. The hearing-threshold and MCL measurements were followed by a series of two-alternative forced-choice (2AFC) interaural-comparison tests. The main test was an interaural pitch-comparison test. This test was preceded by an interaural loudness-comparison test. The goal of the preliminary loudness-comparison test was to collect data that would be used to individually adjust the stimulus level in the left and right ears so as to reduce loudness differences between the two ears; this was done to limit any influence of interaural loudness differences on pitch comparisons across the two ears. In both tests, the participant heard two consecutive pure-tones (each 500-ms in duration including 50-ms on and off linear ramps, separated by a 200-ms silent gap) on each trial. One tone was presented to the right ear, the other tone was presented in the left ear.

For the loudness-comparison test, the two tones had the same frequency (*f*), and the participant was instructed to indicate which of the two tones presented on a trial (one in the right ear, one in the left ear) was louder than the other. Participants’ responses were recorded by the experimenter on a computer using a screen interface with two response buttons labeled ‘1’ and ‘2’. The level of the tone in one ear (hereafter referred to as the ‘reference’ ear) was fixed at 70% of the dynamic range (defined as the difference between the hearing threshold and the MCL) for the current test frequency (*f*). The level of the tone in the contralateral ear (the ‘comparison’ ear) was adjusted using an adaptive one-down, one-up procedure. The procedure stopped after two reversals in the direction of the change in level. The adaptive procedure was run twice, first, with the tone in the comparison ear starting at a higher level than the tone in the reference ear, and a second time, with the converse situation. The mean level at the reversal points across these two runs of the procedure was used to determine the level of the comparison tone needed for this tone to have approximately the same loudness as the reference tone. This loudness-comparison test was performed at three frequencies: *f*_*ref*_, *f*_*ref*_ –1/4 octave, and *f*_*ref*_ +1/4 octave, where *f*_*ref*_ denotes the ‘reference’ frequency. The reason for testing– 1/4 octave and +1/4 octave around *f*_*ref*_ is that these span the frequency range around *f*_*ref*_ tested in the subsequent pitch-comparison test; this made it possible to determine the level needed for the comparison tone to have approximately the same loudness as the reference tone, for all comparison-tone frequencies tested in the pitch-comparison test.

Once this loudness-comparison test was completed, the participant performed an interaural pitch-comparison test. For this test, the participant was instructed to indicate which of the two tones presented on a trial (one in the left ear, one in the right ear) had a higher pitch than the other. Participants’ responses were entered by the experimenter on a computer using a visual interface with two buttons labeled ‘1’ and ‘2’. The level of the reference tone was fixed at 70% of the dynamic range (as in the preceding loudness-comparison test), while the level of the comparison tone was adjusted based on the results of the preceding loudness-comparison test so that, on average, the loudness of the comparison tone was approximately equal to the loudness of the reference tone. In order to further limit any systematic influence of loudness differences on the participant’s responses in the pitch-comparison test, the level of the comparison tone varied randomly across trials over a 10-dB range centered on the nominal level. The frequency of the reference tone was fixed. The frequency of the comparison tone was varied across trials over a half-octave range (+/- 1/4 octave) in 1/16-octave steps. Each combination of reference and comparison frequency was presented 10 times to each participant, in random order.

The above-described combination of loudness- and pitch-comparison tests was performed at different reference frequencies, *f*_*ref*_’s. For the NH participants, *f*_*ref*_’s of 500, 1000, 2000, and 4000 were tested. For HI participants, two *f*_*ref*_’s were tested: 500 Hz, and either 3000 Hz (for participants who had an approximately flat audiogram, with no well-defined edge frequency), or Fc (for participants who had high-frequency hearing loss with a well-defined edge frequency). Fc was equal to 750 Hz for two HI participants, to 1000 Hz for 6 HI participants, to 1500 Hz for 10 HI participants, to 2000 Hz for 19 HI participants, to 3000 Hz for 5 HI participants, and to 4000 Hz for one HI participant. The 500-Hz *f*_*ref*_ was included because, for such a low frequency, there is psychoacoustic evidence that human listeners can make use of temporal fine-structure information for perceptual discrimination [26]. According to pure temporal models wherein pitch perception depends on the fine timing of spikes [15], there should be no binaural diplacusis at such a low frequency. The rationale for testing binaural diplacusis at Fc in HI participants was that this was the highest frequency for which thresholds in the ‘better’ ear had not yet decreased sharply. For HI participants, the reference sound was always presented in the ‘better’ ear (as defined above). For NH participants, the left ear and the right ear were each used as the reference ear, in different blocks of trials.

### Data analysis

**Fig 2:**
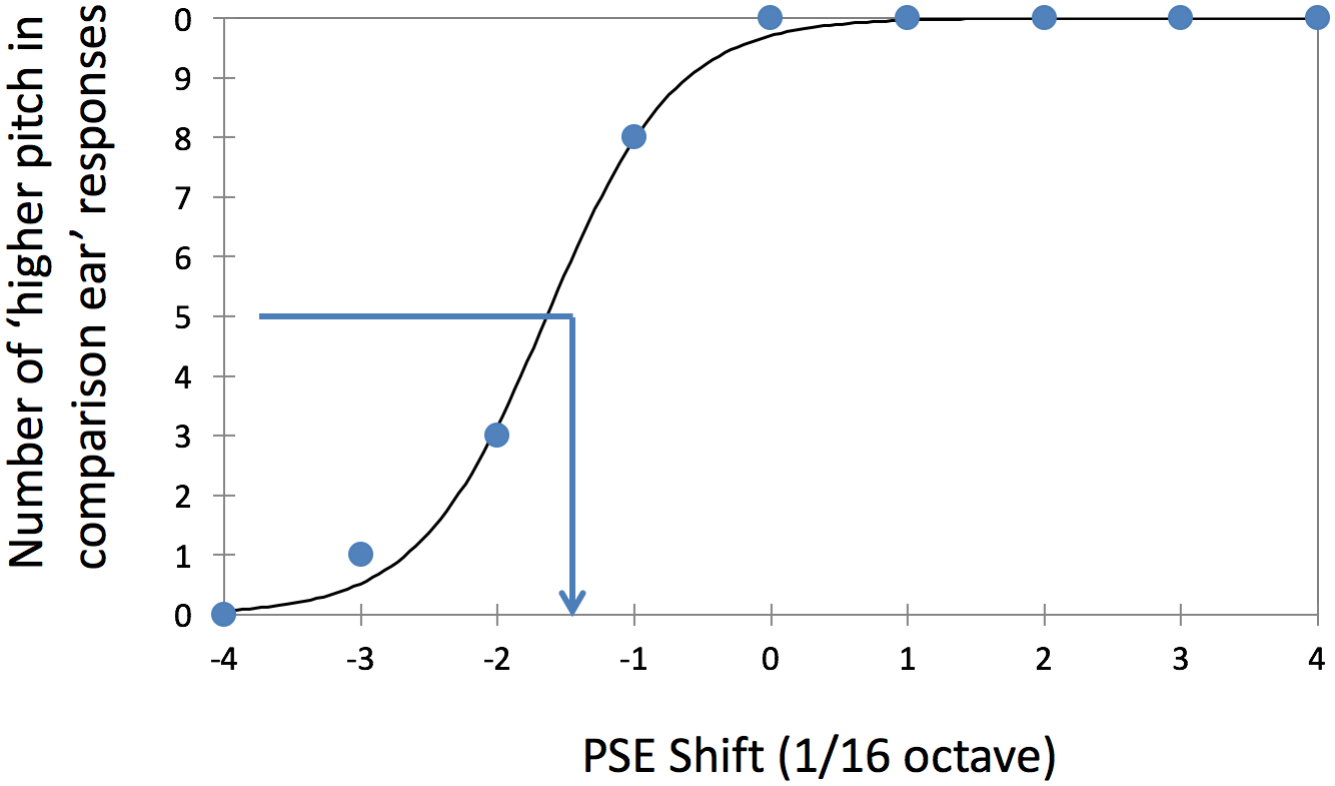
Example psychometric function fitted to data from one NH participant. For this example, the PSE shift was approximately equal to -1.7/16 octave (approximately 7.64%).

For each combination of reference and comparison sound frequency, the number of trials for which the participant indicated perceiving the comparison sound as having a higher pitch than the reference sound was computed. These count data were fitted using a logistic regression model with a binomial error distribution. Fig 2 shows an example of psychometric function fitted to data obtained in an NH participant tested using an *f*_*ref*_ of 1000 Hz. These fits were used to extract the point of subjective equality (PSE), defined as the frequency for which the psychometric function reaches 50%, which corresponds to the frequency for which the comparison tone should be perceived as having the same pitch as the reference sound. These PSEs were used, in turn, to compute PSE shifts, defined as the difference between the PSE and *f*_*ref*_. The PSE shifts reported below are expressed in units of 1/16 octave, the stepsize used in the pitch-comparison test.

To assess whether the PSE shifts differed significantly from zero (no shift), we computed 95% credible intervals (aka as Bayesian confidence intervals) for each PSE shift. This was done using Markov-Chain Monte Carlo algorithms implemented in the OpenBUGS software [27].

### Material

All tests were performed with the participants in an audiometric testing cabin. Hearing thresholds were measured using either an Aurical (Gn Otometrics) or an Affinity (Interacoustics) system with Sennheiser HD 202 headphones. For the pitch-perception tests, stimuli were generated and presented using Matlab. They were converted to analog signals (24 bits; 44.1 kHz sampling frequency) using a SoundBlaster X-FI HD SB1240 CREATIVE sound card, and delivered to the participants’ ears through Sennheiser HD 202 headphones.

## Results

### NH participants

**Fig 3:**
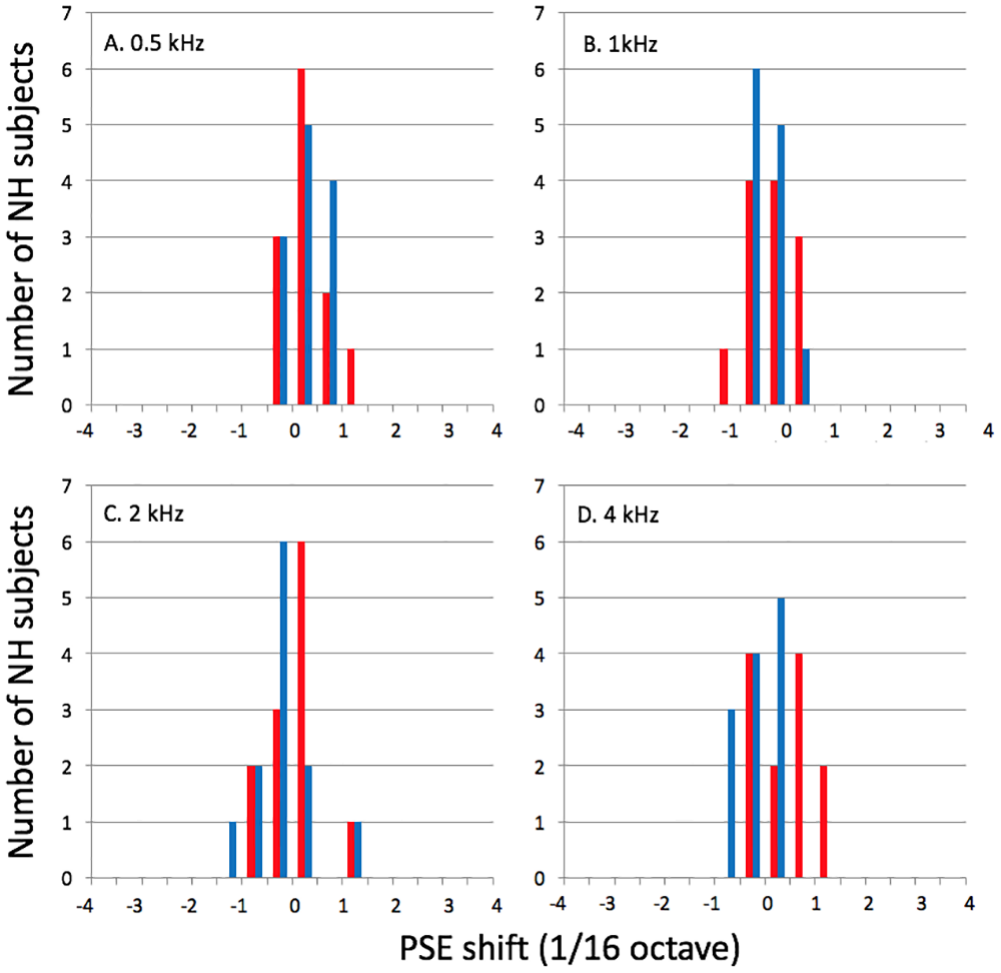
Distribution of PSE shifts for NH participants. The different panels (A-D) show data for different reference frequencies. Red bars: right ear; blue bars: left ear. These distributions were computed using bins of 1/32 octave.

Fig 3 shows PSE-shift distributions for the NH participants. The four panels correspond to the four base frequencies, and within each panel, the left- and right-ear data are shown separately (using different colors). For this group, a PSE shift larger than 1.5/16 octave (about 7%) was observed for only one listener, in one test condition (2-kHz *f*_*ref*_, left ear). Therefore, when analyzing the data of the HI listeners, pitch shifts smaller than 1.5/16 octave (about 7%) may be regarded as ‘normal’.

When looking at these individual data, an important question, is whether any of these pitch shifts is statistically significant, or whether all of these shifts can be confidently imputed to measurement error. To address this question, we examined 95% Bayesian confidence intervals for the individual pitch shifts. These intervals were computed with the assumption that the count data that were used to fit psychometric functions followed a binomial distribution. Whenever the 95% confidence intervals computed in this way did not include zero, we concluded that the pitch shift measured in the considered participant and test condition could not be imputed to limited precision of the psychometric procedure used. Using this approach, we found that 23 out of the 96 pitch shifts (or about 24%) measured in these NH participants could not be imputed simply to measurement error.

**Fig 4:**
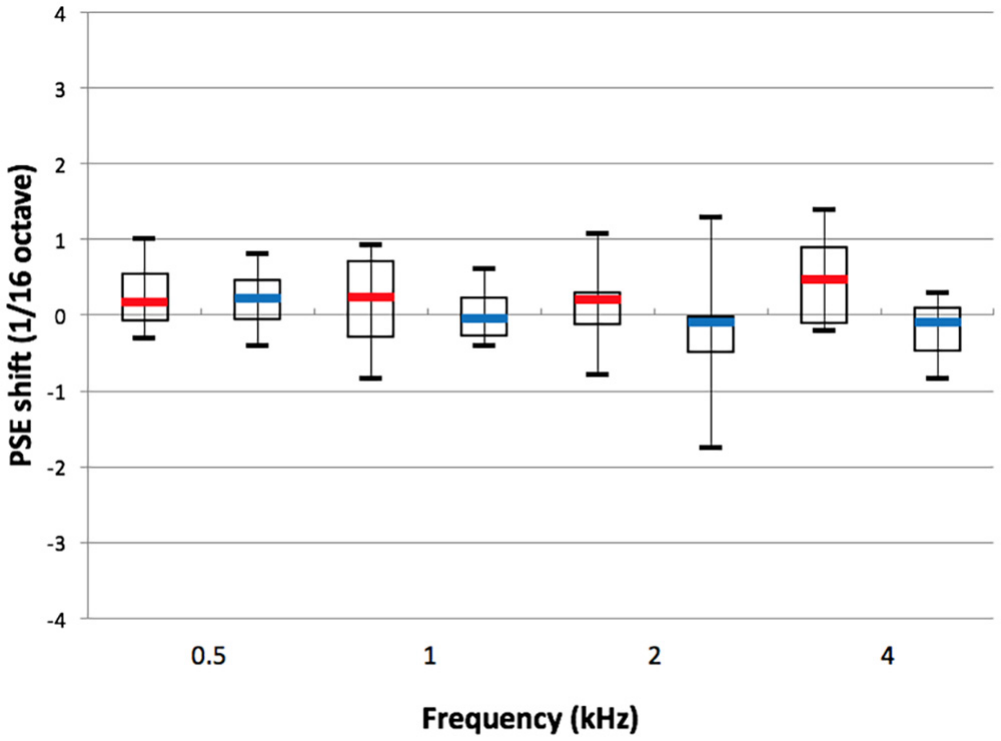
Mean PSE shifts for the NH group. Red: right ear; blue: left ear. Test frequencies are listed on the abscissa.

In order to determine whether PSE shifts varied significantly as a function of the test frequency, or to differ between the left and right ears, we computed mean PSE shifts and their associated 95% Bayesian confidence intervals. The results are shown in Fig 4. The whiskers show 95% Bayesian confidence intervals around the mean. All of these intervals span zero, indicating that none of the mean pitch shifts departed significantly from zero. Given this outcome, further exploring differences across frequency or between the two ears was moot.

### HI participants

**Fig 5:**
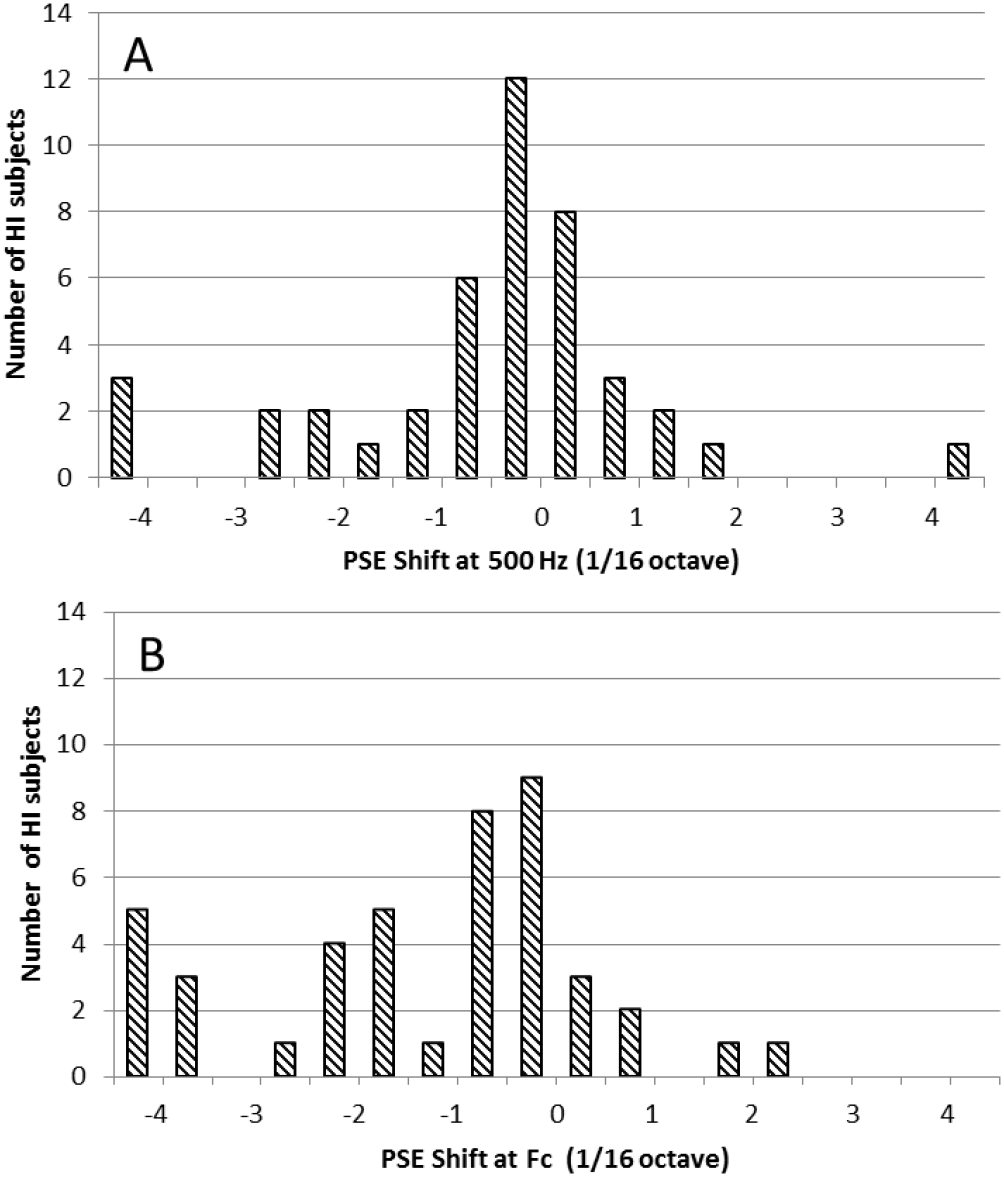
Distributions of PSE shifts for HI participants. A: Data for the 500-Hz reference frequency. B: Data for the reference frequency = Fc.

Fig 5 shows the distributions of PSE shifts measured at 500 Hz and at Fc in the HI participants. Looking at the 95% Bayesian confidence intervals for individual PSE shifts, we found that 24 and 28 (out of 43) HI participants had significant PSE shifts at 500 Hz and Fc, respectively. Of these, 19 participants had significant PSE shifts at both frequencies. For 29 of the HI participants, the statistical outcome was consistent across the two test frequencies, showing either no PSE shift at both frequencies or a shift at both frequencies.

Using 1.5/16 octave (or 7%) as a normative boundary, we found that 10 out of 43 HI participants had abnormally large pitch shifts at 500 Hz, while 20 out of 43 had abnormally large shifts at Fc. For a vast majority of these subjects (8 out of 10, and 18 out of 20, respectively), the direction of the shift was consistent with pitch being perceived as higher on the side with larger (i.e., ‘worse’) hearing thresholds than on the opposite side.

Note that, although the range used to measure psychometric functions in the interaural pitch-comparison test was limited to +/- 1/4 octave, pitch shifts larger than 1/4 octave could be, and sometimes were, obtained using logistic-regression analysis. These large pitch shifts must be interpreted cautiously, since they are based on extrapolation beyond the actual range of the data.

**Fig 6:**
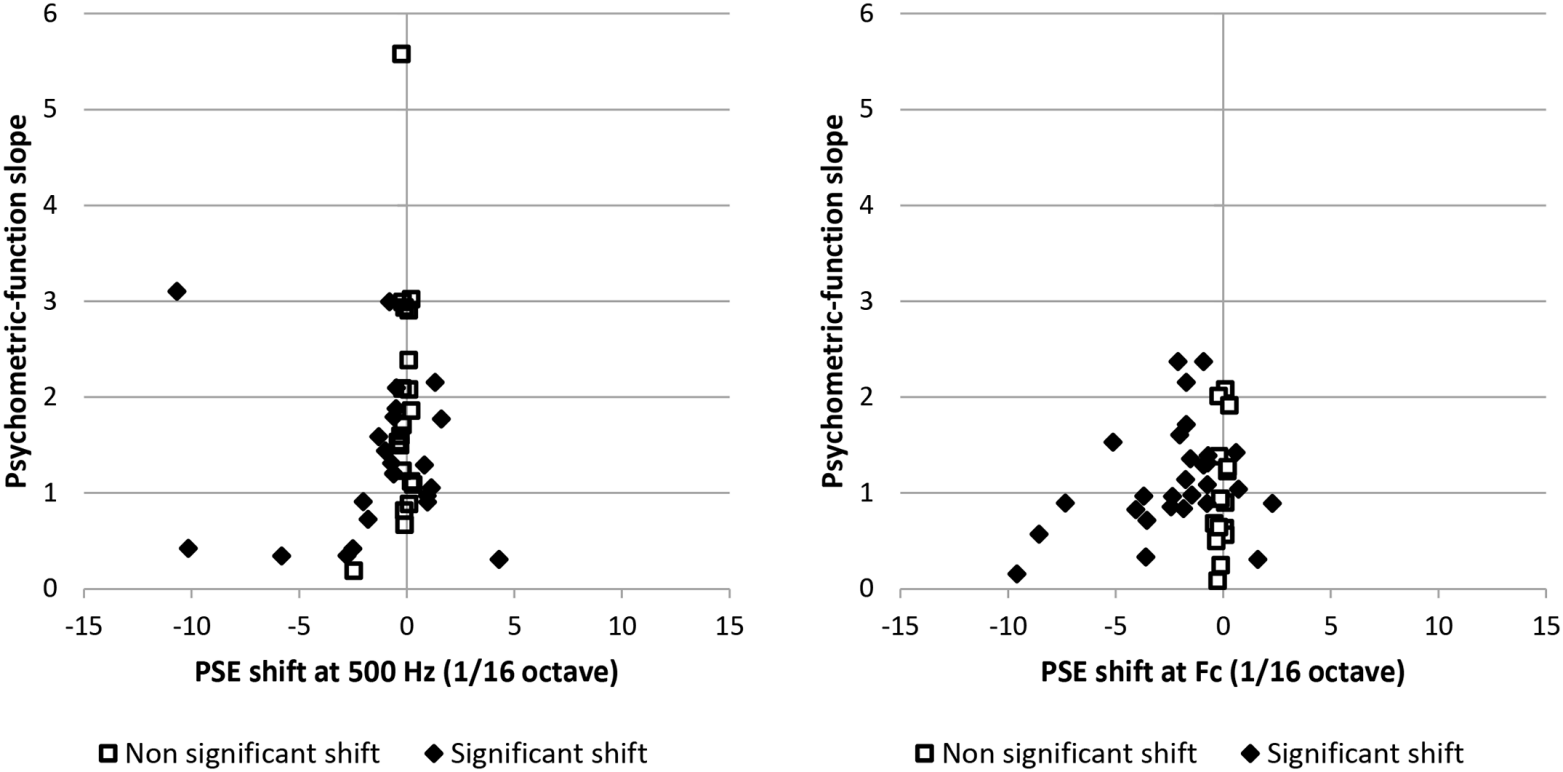
PSE shift versus the psychometric-function slope in HI participants. A: Data for the 500-Hz reference frequency. B: Data for the reference frequency = Fc.

To determine whether the pitch shifts that were not statistically significant could be accounted for by shallow psychometric-function slopes, indicating poor or highly variable interaural pitch-matching abilities, we examined the relationship between the psychometric-function slope and the pitch shifts. Fig 6 shows a scatterplot of the pitch-shifts versus the psychometric-function slope for the two test frequencies (500 Hz and Fc) in the HI participants. Inspection of this figure reveals that small and non-significant pitch shifts were not systematically associated with shallow psychometric-function slopes. Therefore, the absence of significant binaural diplacusis in some HI participants cannot be imputed solely to poor or highly variable pitch-matching abilities in these participants. In fact, the psychometric-function slopes of the HI participants were not statistically different from those of the NH participants (two-sample t-test on the log-transformed slopes, t = 1.27, df = 53, p = 0.21). The geometric-mean slope was equal to 1.28 (95% confidence interval = [0.31;5.23]) for the HI participants, and to 1.70 (95% confidence interval = [0.62; 4.62]) for the NH participants.

**Fig 7:**
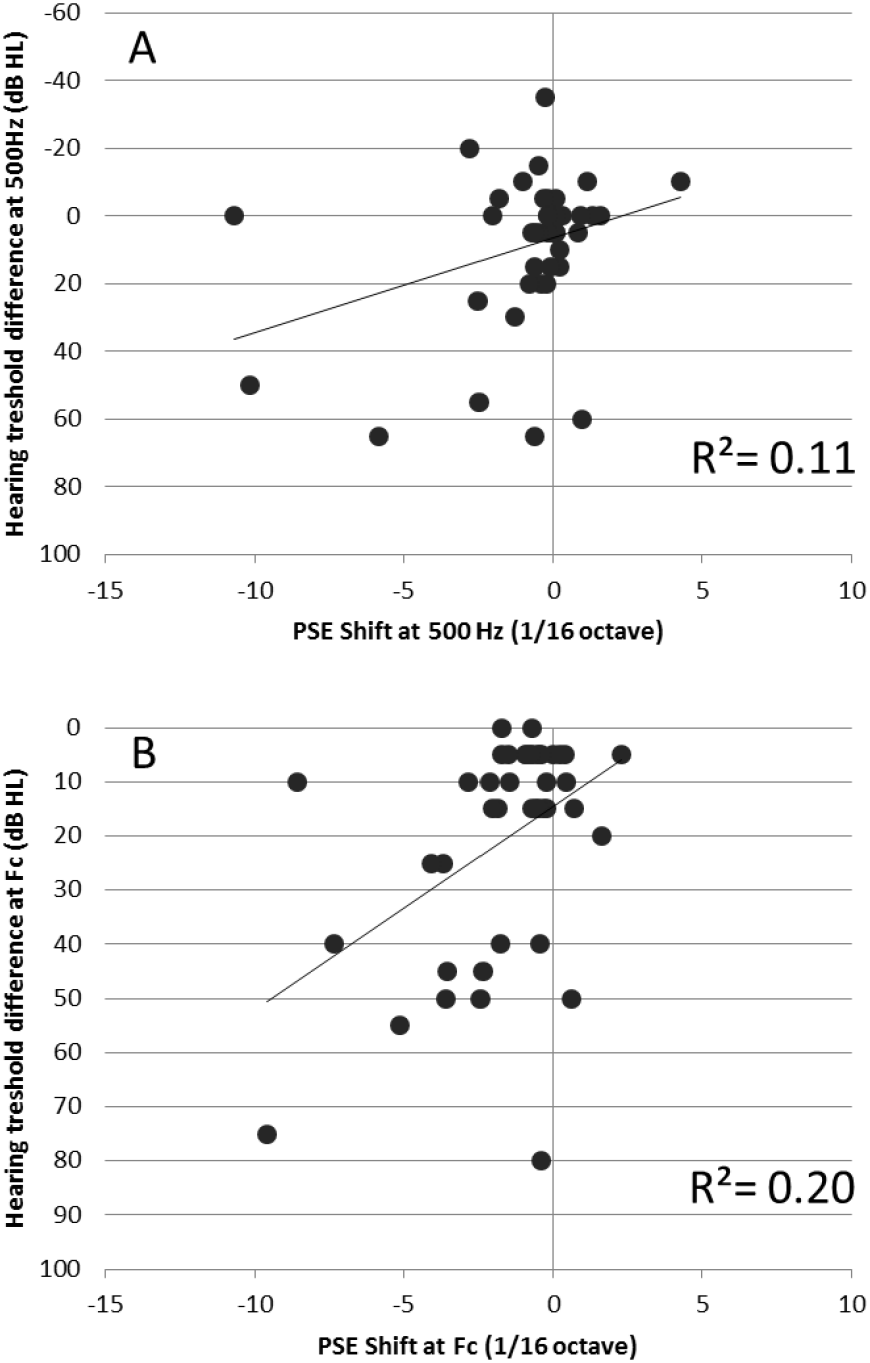
PSE shift versus hearing-threshold asymmetry in HI participants. Each data point corresponds to one individual.

Fig 7 shows a scatter plot of the pitch shifts at 500 Hz and Fc versus the left-right difference in hearing thresholds at 500 Hz and Fc, respectively. For both frequencies, a significant negative correlation was found, such that the larger the asymmetry in hearing thresholds, the more negative the pitch shift; negative pitch shifts correspond to a higher perceived pitch on the side with the higher (i.e., ‘worse’) hearing thresholds. For 500 Hz, the Pearson correlation coefficient was -0.33 (*p* = 0.03); for Fc, the Pearson correlation coefficient was -0.45 (*p* = 0.003). A significant correlation was also found between the pitch shifts at 500 Hz and the pitch shifts at Fc (*r* = 0.50, p = 0.001). However, it might be argued that the correlation at Fc is driven to a large extent by one data point, from the participant who had a large negative estimated pitch shift (about 3/4 octave). When this data point was removed, the correlation became subsantially lower, and was no longer statistically significant (*r* = -0.30, p = 0.05).

## Discussion

### Distribution of binaural diplacusis in NH and HI individuals

Results of previous studies suggest that binaural diplacusis can be observed in individuals with NH, although the effect usually remains small [20–30]. The results of the present study are generally consistent with this. Here, using an individual statistical criterion for measurement error (95% Bayesian confidence intervals computed using a logistic regression model with binomially distributed errors), statistically significant pitch shifts between the left and right ears were found for about one fourth of the pitch-comparison tests performed in NH participants. However, with one exception, the pitch shifts measured in NH participants were quite small: less than 1.5/16 octave (about 7%). Therefore, it appears that binaural diplacusis effects less than 7% (approximately, 1 semitone on the musical scale) can be considered as ‘normal’.

Also consistent with earlier findings, the results of the present study indicate that binaural diplacusis is more prevalent, and the magnitude of the effect is often larger, in HI participants than in NH participants. For the test condition in which the reference frequency corresponded to the cutoff frequency (Fc) of the audiogram, approximately two thirds of the HI participants showed statistically significant interaural pitch shifts, and for about half of the HI participants, the pitch shifts were abnomally large, i.e., larger than 1.5/16 octave (about 7%). These proportions were somewhat lower for the 500 Hz test frequency; however, even for this low frequency, about one fourth of the HI participants displayed abnormally large interaural pitch shifts.

The lack of statistically significant binaural diplacusis in some of the HI participants might be due to these individuals having poor or highly variable interaural pitch-matching abilities, which should be reflected in shallower psychometric-function slopes in the interaural pitch-comparison task for these participants than for other participants. In particular, based on data from previous studies showing poorer or more variable performance in monaural or interaural pitch-comparison tasks for HI listeners than for NH listeners [5], poorer-than-normal interaural pitch-matching abilities could be expected *a priori* for the HI participants. However, we found no statistically significant difference in the slopes of the psychometric functions for the interaural pitch-comparison task between the HI and the NH participants. Moreover, the small and statistically non-significant pitch shifts in the HI participants were not systematically associated with shallow psychometric-function slopes. Thus, we may conclude that, at least for some HI participants, the absence of significant binaural diplacusis is not due merely to a poor or highly variable interaural pitch-matching ability.

Based on the findings of earlier studies, we expected the magnitude of binaural diplacusis to increase with the interaural asymmetry in hearing thresholds. The results are partly consistent with this expectation, in that significant correlations were found between the interaural difference in hearing thresholds and the left-right pitch shifts for both test frequencies (500 Hz and Fc). However, these correlations were relatively low, with the interaural asymmetry in hearing thresholds accounting for only 22% of the variance in the interaural pitch shifts, at most. Moreover, the correlations were not very robust; for the condition in which the reference frequency was equal to Fc, the correlation vanished after one participant, who was possibly an outlier, was removed. These results suggest that binaural diplacusis depends on other factors, besides factors related to the interaural asymmetry in hearing thresholds. At present, these other factors remain poorly understood. They could stem from peripheral and/or central modifications in auditory function, which accompany hearing loss, but which do not manifest as shifts in absolute thresholds (see next section).

Interesting, for the vast majority of the HI participants, the direction of the pitch shifts was consistent with pitch being perceived as higher on the side on which hearing thresholds were higher (‘worse’), compared to the contralateral side (‘better’ ear). This finding provides some constraints on the nature of the underlying biological mechanisms of binaural diplacusis, as discussed in the following section.

### Possible biological mechanisms and implications for pitch theories

An effect, which could have contributed to the results of this study, relates to shifts in pitch with sound intensity. Specifically, previous studies have found that, as stimulus intensity increases, the pitch of pure tones tends to shift toward higher frequencies above 1 kHz, and toward lower frequencies below 1 kHz [31–32]–although, with substantial interindividual variability and, in some cases, nonmonotonic pitch-intensity functions [33]. However, such intensity-dependent pitch shifts are unlikely to entirely explain the results of this study because, firstly, they are usually quite small, with a maximal pitch shift of about 4% for an 80-dB change in sound level [32]. Secondly, as indicated above, the direction of pitch shifts related to stimulus intensity has been found to switch below 1 kHz, whereas in the present study, the direction of pitch shifts was usually the same at 500 Hz as at higher frequencies, including frequencies higher than 1 kHz. Thus, while we cannot entirely rule out the possibility that small pitch shifts related to differences in sound intensity across the two ears may have been present in some of the tests performed in the present study, such level-dependent pitch shifts cannot be the main explanation for the findings.

Another effect, which may have contributed to the results of this study, relates to central neural plasticity. Several studies have shown cortical reorganization of tonotopic maps following peripheral damage [34–38]. Specifically, following cochlear lesions, neurons in primary auditory cortex that were formerly excited maximally by tones having frequencies corresponding to the damaged cochlear region, now have their best frequencies shifted toward frequencies outside, or on the border of, the damaged region [34–38]. Such central-reorganization effects could result in upward shifts in pitch following high-frequency cochlear damage: after the damage, neurons that used to code high frequencies (above the audiogram cutoff frequency) start responding to sounds having lower frequencies; as a result, the tonotopic pattern of excitation evoked by stimuli with frequencies near, or below, the audiogram cutoff is shifted toward higher frequencies than before the damage. However, a problem with this explanation is that, in the current study, binaural diplacusis was observed, not only at the audiogram edge frequency, but also at 500 Hz, which for many participants, was one octave or more *below* the audiogram cutoff frequency. Although the effect was a little less prevalent at 500 Hz than at Fc, the fact that it was observed at 500 Hz as well, is difficult to reconcile with a central-reorganization model in which central reorganization occurs only at, or near, the region in the tonotopic map that corresponds to the audiogram edge.

An alternative explanation for these results is in terms of changes in cochlear mechanics following cochlear damage. Physiological observations indicate that, following cochlear damage, the peak of excitation on the basilar membrane shifts toward a more basal region [39]. A corollary of this effect is a shift in the peak of the neural tuning curves of auditory neurons toward lower frequencies [18]. If the mechanism for decoding pitch implemented in the central auditory system involves a place code, whereby a given place within the central tonotopic map always corresponds to the same pitch, the pitch shifts measured in the present study could be explained as a consequence of basal shifts caused by cochlear damage impacting the outer hair cells. Consistent with this explanation, we found that for a vast majority of the HI participants, the pitch shifts were positive, indicating that pitch was perceived as higher on the side in which hearing thresholds were higher, i.e., ‘worse’, than on the opposite side. Interestingly, studies comparing the normal ‘anatomical’ cochlear map with the ‘physiological’ cochlear map following extensive damage of the cochlear active mechanism estimated the magnitude of the basal shift at 0.3–0.5 octave in mammals [39]. The pitch shifts that were measured in HI participants in the current study usually fall within this range.

One limitation of the damage-induced basal-shift explanation outlined in the previous paragraph stems from the fact that not all of the interaural pitch shifts measured in this study were in the same direction. Specifically, two HI participants showed *positive* abnormal (>1.5/16 octave, or about 7%) pitch shifts, indicating a higher pitch on the side with the lower (i.e., ‘better’) hearing thresholds than on the opposite side. For one participant, the positive pitch shift was observed in the condition where *f*_*ref*_ was equal to 500 Hz; for the other participant, it occurred in the condition where *f*_*ref*_ was equal to Fc. These positive pitch shifts may have been mediated by a different mechanism than the damage-induced basal shift observed in the physiological studies cited above. Some studies have found *upward* shifts in the best frequency of auditory nerve fibers with increasing stimulus level for frequencies lower than about 1 kHz, contrasting with *downward* shifts for higher test frequencies [40–41]. It is not clear whether these frequency-and-level-dependent shifts are related to best-frequency shifts induced cochlear damage. Importantly, for the majority of the HI participants in the present study, the interaural pitch shifts were in a direction consistent with a basal shift in functional cochlear maps (relative to the normal cochlear map), and with downward shifts in the best frequency of auditory neurons.

An extreme form of peripheral damage corresponds to a ‘dead cochlear region,’ which is a region of the cochlea with no residual functional hair cells or auditory-nerve fibers. Using pitch matches across the two ears, and monaural octave matches in HI listeners with cochlear dead regions, a previous study found that pure tones with frequencies falling inside a dead region evoked an unclear and/or abnormal [42]. Although we did not assess cochlear dead regions in the present study, previous studies in which dead regions were assessed indicate that these are usually associated with pure-tone hearing thresholds larger than 90 dB HL, or 75–80 dB HL at low frequencies [43]. In the present study, two participants had hearing thresholds larger than 75 dB HL at 500 Hz; both of these participants showed statistically significant binaural diplacusis at this frequency. Four participants had hearing thresholds larger than 75 dB HL at Fc; of these four participants, three had statistically significant binaural diplacusis at Fc. It is possible that, for these listeners, the binaural diplacusis was related to cochlear dead regions. However, considering that the majority of participants in the study showed significant binaural diplacusis even though their hearing threshold were all lower than 75 dB HL, cochlear dead regions do not appear to be a necessary condition for binaural diplacusis.

While the results of the present study are, for the most part, consistent with an injury-induced basal-shift in the cochlear mechanics, they support the view that pitch perception depends on a place code [1–2]. However, based on different datasets, other authors have reached a different conclusion. In particular, in some studies, the interaural pitch shifts were found to be considerably less than expected based on measurements of psychoacoustic tuning curves, which indicated different tonotopic representations of pitch in the left and right ears of these listeners [9–11]. For example, Turner et al. (1982) measured pitch shifts of about 5% at 250 Hz in a listener with low-frequency hearing loss, the psychoacoustic tuning curves data of whom indicated that pure-tones with frequencies lower than 500 Hz were all mapped to the cochlear place corresponding to 500 Hz. The only way to reconcile the findings of the current study with those of previous studies, such as Turner et al.’s, is to consider that interaural pitch shifts can result from different mechanisms, depending on individual factors related to cochlear damage and/or central effects.

Nonetheless, in the current state of knowledge, the results of the present study and those of previous studies of binaural diplacusis are difficult to reconcile with purely temporal theories of pitch coding, according to which pitch is encoded in the fine timing of neural spikes evoked by the stimulus [16;44–46]. With one exception [19], studies that have looked for alterations of temporal response patterns in single auditory-nerve fibers following drug- or noise-induced cochlear damage have not found any, at least, for sounds presented in quiet [20–24]. Based on these physiological findings, therefore, one would not expect binaural diplacusis if pitch depends on a purely temporal code. However, the results of this study and of previous studies may be explainable in terms of a spectrotemporal model of pitch coding, in which pitch perception depends on a combination temporal and place cues [47–49].

## Supporting Information

S1 Table OpenBUGS code. OpenBUGS code for the Bayesian model used analyze the pitch-comparison data of the normal-hearing and hearing-impaired participants
(XLSX)

S2 Table Data of the normal-hearing and hearing-impaired participants. (DOCX)
