# Supplementary material for: Binaural Diplacusis and Its Relationship with Hearing-Threshold Asymmetry
Source: PLoS One. 2016 Aug 18;11(8):e0159975. doi: 10.1371/journal.pone.0159975 (PMC4990190; doi:10.1371/journal.pone.0159975)
Supplement: S2 Table — (DOCX) [file pone.0159975.s002.docx]

**S1. OpenBUGS code for the Bayesian model used analyze the pitch-comparison data of the normal-hearing and hearing-impaired participants**

model{

# Model used to analyze the data of the normal-hearing listeners

for (freq in 1:4){ # Loop through reference frequencies

dttotm[freq] <- ttotm[freq,2] - ttotm[freq,1]

dsm[freq] <- sm[freq,2] - sm[freq,1]

for (side in 1:2){ # Loop through test sides (left, right)

ttotm[freq,side] <- mean(ttot[freq,side,])

logsm[freq,side] <- mean(logs[freq,side,])

sm[freq,side] <- exp(logsm[freq,side])

for (j in 1:nj){ # Loop through subjects

ttot[freq,side,j] ~ dnorm(0, 0.0150) # Threshold of logistic function used to compute probability of 'higher pitch in comparison ear' response

logs[freq,side,j] ~ dnorm(0.55, 1.25)

s[freq,side,j] <- exp(logs[freq,side,j]) # Slope of logistic function used to compute probability of 'higher pitch in comparison ear' response

for (i in 1:ni){ # Loop through comparison-tone frequencies

# Probability of 'higher pitch in comparison ear' response is modeled as a logistic function of the comparison-tone frequency

p[freq,side,j,i] <- 1/(1+exp(-s[freq,side,j]*(x[i]-ttot[freq,side,j])))

# Number of 'higher pitch in comparison ear' responses modeled as a binomial

n[freq,side,j,i] ~ dbin(p[freq,side,j,i], 10)

}

}

}

}

}

# Data file used as input into model for analyzing the data of the normal-hearing participants

list(

ni = 9,

nj = 12,

x = c(-4, -3, -2, -1, 0, 1, 2, 3, 4),

n=structure(

.Data=c(

0,0,1,3,8,7,8,9,10,0,0,0,0,4,9,9,10,10,0,0,0,1,4,10,10,10,10,0,0,1,1,4,9,10,9,10,0,0,0,0,5,7,8,9,10,0,0,0,1,7,9,10,10,10,0,0,0,1,3,9,10,10,10,0,0,0,1,5,6,10,9,10,0,0,0,1,4,6,9,9,10,0,1,0,0,7,10,10,10,10,0,0,0,0,2,7,7,9,10,0,0,0,0,8,7,9,10,10,0,1,2,4,4,8,9,10,10,0,0,0,0,5,10,10,10,10,0,0,0,0,6,10,10,10,10,0,0,0,1,3,9,9,10,10,0,0,0,1,2,6,9,9,10,0,0,1,0,4,9,10,9,10,0,0,0,1,3,8,8,10,10,0,0,1,3,6,9,10,10,10,0,0,3,5,6,3,9,8,9,0,0,0,0,2,8,10,10,10,0,0,2,2,1,8,7,9,10,0,1,1,2,6,6,8,9,10,1,0,0,1,3,9,9,8,10,0,1,1,3,6,8,10,10,10,0,0,0,0,3,9,10,10,9,0,0,0,2,3,8,10,10,10,0,1,0,0,3,5,8,10,10,0,0,0,0,2,10,10,10,10,1,1,0,0,8,8,9,9,10,0,0,3,2,5,9,7,10,10,0,0,0,1,3,6,7,9,10,0,0,0,3,10,10,10,10,10,0,0,1,0,2,6,9,10,8,1,3,1,2,5,8,10,8,10,0,1,1,2,4,6,9,8,10,0,0,0,0,3,10,10,10,10,0,0,0,0,7,10,10,10,10,1,0,0,3,5,10,10,10,10,0,0,0,0,3,8,8,10,10,0,1,0,0,4,10,10,10,10,0,0,0,1,4,9,10,10,10,0,0,1,2,6,10,10,10,10,0,0,4,2,4,6,10,10,10,0,0,0,0,2,10,10,10,10,0,1,0,3,6,8,8,10,10,0,0,3,4,4,8,10,10,9,0,1,0,1,4,6,7,6,10,0,0,0,0,3,8,10,10,10,0,0,0,0,2,10,10,10,10,0,1,0,1,4,8,9,10,10,0,0,0,1,6,8,10,10,10,0,1,0,2,8,10,10,10,9,1,0,0,4,9,10,9,10,10,0,1,4,0,3,9,9,10,10,1,0,2,3,5,6,9,10,10,0,0,0,0,4,9,10,10,10,1,1,1,1,3,9,7,10,10,0,1,1,0,2,8,10,10,10,1,0,2,1,4,8,9,10,10,0,0,0,1,5,10,10,10,10,0,0,0,3,7,10,10,10,10,1,0,0,4,3,8,10,10,10,0,0,0,0,6,10,10,10,10,0,0,0,1,6,10,9,10,10,0,0,3,2,6,10,9,10,10,0,0,0,4,5,10,10,10,10,3,4,1,7,7,9,10,10,10,0,0,0,0,3,9,10,10,10,0,0,0,0,0,5,8,10,9,1,1,1,1,8,10,9,8,10,0,0,0,0,6,5,7,8,9,0,0,0,0,4,7,10,10,10,0,0,0,0,6,10,10,10,10,0,0,0,0,7,9,10,10,10,0,0,0,1,7,7,9,9,10,0,0,1,1,4,10,10,10,10,0,0,0,0,3,8,9,9,9,0,0,0,3,3,7,7,10,10,1,2,2,2,3,9,10,9,9,0,0,0,0,0,6,10,10,10,0,3,1,1,3,6,8,9,4,0,0,0,0,4,6,8,9,10,2,3,1,2,3,7,9,10,10,0,0,0,0,5,10,10,10,10,0,0,0,0,5,10,10,10,10,1,0,1,2,1,9,10,10,9,0,0,0,0,9,10,10,10,10,0,1,2,0,6,9,10,10,10,1,3,1,1,4,10,10,10,10,0,0,0,4,4,9,8,10,9,3,2,2,4,6,6,10,10,9,0,0,0,0,4,10,10,10,10,0,0,0,0,4,9,10,9,10,1,0,3,3,5,10,10,10,10),

.Dim=c(4,2,12,9))

)

model{

# Model used to analyze the data of the hearing-impaired listeners

for (j in 1:nj){

t[j] ~ dnorm(0, 0.0150) # Threshold of logistic function used to compute probability of 'higher pitch in comparison ear' response

logs[j] ~ dnorm(0.55, 1.25)

s[j] <- exp(logs[j]) # Slope of logistic function used to compute probability of 'higher pitch in comparison ear' response

for (i in 1:ni){ # Loop through comparison-tone frequencies

p[j,i] <- 1/(1+exp(-s[j]*(x[i]-t[j]))) # Probability of 'higher pitch in comparison ear' response is modeled as a logistic function of the comparison-tone frequency

n[j,i] ~ dbin(p[j,i], 10) # Number of 'higher pitch in comparison ear' responses modeled as a binomial

}

}

}

# Data file used as input into model for analyzing the data of the hearing-impaired participants for *f_ref_* = 500 Hz

list(

ni = 9,

nj = 43,

x = c(-4, -3, -2, -1, 0, 1, 2, 3, 4),

n=structure(

.Data=c(

0,0,0,0,4,10,10,10,9,0,0,1,1,1,6,7,8,10,0,0,1,4,6,10,10,10,10,0,0,0,0,5,9,10,10,10,1,0,2,2,7,10,9,10,10,0,0,0,3,4,10,10,10,10,0,0,0,0,6,8,10,10,10,0,0,2,5,6,10,9,10,10,0,0,1,2,8,9,9,10,10,0,0,0,1,5,10,10,10,10,0,1,2,4,9,9,10,10,10,1,1,1,2,2,4,5,3,4,0,0,0,0,0,4,9,9,10,0,0,1,2,3,5,6,10,9,0,0,0,2,3,9,10,10,10,0,0,0,3,5,10,9,10,10,0,1,1,9,7,10,10,10,10,0,3,1,4,3,6,9,10,10,10,10,10,10,10,10,10,10,10,0,0,0,1,0,2,6,10,10,4,6,6,5,7,5,9,9,10,0,0,1,1,7,6,7,10,10,1,4,5,7,7,10,10,10,10,0,0,0,3,8,9,10,10,10,0,0,0,0,8,10,10,10,10,9,10,10,9,10,10,10,9,10,0,0,1,2,10,10,10,10,10,1,1,0,0,4,8,10,9,10,0,1,1,3,4,8,8,10,9,0,0,1,3,6,10,10,10,10,0,0,1,2,6,10,10,9,10,0,0,0,1,6,10,10,10,10,0,0,0,0,4,9,10,10,10,0,0,0,1,3,6,8,9,10,0,1,0,3,2,3,7,10,10,0,0,1,3,7,7,9,10,10,2,3,4,7,8,7,10,10,10,1,0,2,4,6,8,7,10,8,0,0,0,4,6,8,10,10,10,4,6,6,5,7,5,5,10,6,6,7,8,8,9,10,10,8,9,4,4,4,7,8,9,8,9,9,0,1,1,5,6,8,10,10,10),

.Dim=c(43,9))

)

# Data file used as input into model for analyzing the data of the hearing-impaired participants for *f_ref_* = Fc

list(

ni = 9,

nj = 43,

x = c(-4, -3, -2, -1, 0, 1, 2, 3, 4),

n=structure(

.Data=c(

0,0,1,4,9,10,9,9,10,0,1,6,9,10,10,10,10,10,0,5,10,5,7,10,10,10,10,1,0,0,1,9,7,9,10,10,4,7,7,9,10,10,10,10,10,2,0,1,1,9,10,10,10,9,0,1,3,8,10,10,10,10,10,2,2,4,3,3,7,10,9,10,0,0,3,5,8,8,10,10,10,0,0,1,4,9,10,10,10,10,0,1,1,4,4,10,10,10,10,0,1,2,2,8,9,10,10,10,0,0,0,0,1,4,5,7,7,8,6,9,9,9,10,8,8,5,0,0,0,1,6,8,10,10,10,0,1,5,6,10,10,10,10,10,10,8,10,10,10,10,10,10,10,0,0,1,3,7,5,10,8,7,9,10,9,10,9,10,10,10,10,0,0,0,3,2,4,10,10,10,3,7,1,5,5,6,5,5,10,0,0,3,3,2,8,8,9,10,2,2,5,6,8,10,9,10,10,0,0,1,2,3,5,8,9,10,0,0,1,2,6,10,10,10,10,7,9,10,10,10,10,10,10,10,1,2,5,5,9,10,10,10,10,0,2,5,9,9,10,10,10,10,1,2,2,2,3,9,10,10,10,1,0,0,0,7,10,10,10,10,8,7,7,5,5,4,4,5,5,0,0,0,1,5,8,8,9,10,2,2,4,3,4,6,9,10,9,1,0,3,7,10,10,9,10,10,1,1,1,7,5,8,9,10,10,2,2,2,5,9,9,10,10,10,6,5,6,7,10,10,10,10,10,4,1,4,3,0,3,6,7,8,2,1,3,6,4,5,9,8,9,1,1,1,4,7,10,9,9,7,5,4,4,9,9,9,9,9,7,2,3,5,9,9,9,10,10,10,5,7,8,8,10,10,10,10,10),

.Dim=c(43,9))

)
